# Exploring the Landscape of Pre- and Post-Synaptic Pediatric Disorders with Epilepsy: A Narrative Review on Molecular Mechanisms Involved

**DOI:** 10.3390/ijms252211982

**Published:** 2024-11-07

**Authors:** Giovanna Scorrano, Ludovica Di Francesco, Armando Di Ludovico, Francesco Chiarelli, Sara Matricardi

**Affiliations:** 1 Department of Pediatrics, University of Chieti-Pescara, Sant’Annunziata Hospital, 66100 Chieti, Italy; scorranogiovanna10@gmail.com (G.S.); armandodl@outlook.com (A.D.L.); francesco.chiarelli@unich.it (F.C.); 2 Department of Neonatology, University of L’Aquila, San Salvatore Hospital, 67100 L’Aquila, Italy; difrancesco.ludovica@gmail.com

**Keywords:** synaptopathies, genetics, neurodevelopment, neurotransmitter release

## Abstract

Neurodevelopmental disorders (NDDs) are a group of conditions affecting brain development, with variable degrees of severity and heterogeneous clinical features. They include intellectual disability (ID), autism spectrum disorder (ASD), attention-deficit/hyperactivity disorder (ADHD), often coexisting with epilepsy, extra-neurological comorbidities, and multisystemic involvement. In recent years, next-generation sequencing (NGS) technologies allowed the identification of several gene pathogenic variants etiologically related to these disorders in a large cohort of affected children. These genes encode proteins involved in synaptic homeostasis, such as SNARE proteins, implicated in calcium-triggered pre-synaptic release of neurotransmitters, or channel subunit proteins, such as post-synaptic ionotropic glutamate receptors involved in the brain’s fast excitatory neurotransmission. In this narrative review, we dissected emerged molecular mechanisms related to NDDs and epilepsy due to defects in pre- and post-synaptic transmission. We focused on the most recently discovered SNAREopathies and AMPA-related synaptopathies.

## 1. Introduction

Neurodevelopmental disorders (NDDs) are a heterogeneous group of conditions, including intellectual disability (ID), autism spectrum disorder (ASD), and attention-deficit/hyperactivity disorder (ADHD). They present great variability in the setting of clinical manifestations, and frequently, a clear genotype–phenotype correlation is not found [1]. Molecular etiopathogenesis is complex, with genetic, epigenetic, and environmental factors involved, and many affected individuals still have an inaccurate diagnosis [2].

In recent years, advances in next-generation sequencing (NGS) technologies have allowed us to understand better the genetic landscape underlying these conditions and their association with epilepsy [3]. The broad application of NGS techniques has helped cluster patients with similar phenotypes worldwide, encouraging genotype–phenotype correlations [4,5,6,7,8,9,10,11]. Some of the most interesting genes involved in NDDs and epileptic seizures are those coding for synaptic proteins, and several studies in the last decade highlighted the central role of the synapse in neurodevelopment and neurotransmission [12,13,14]. Synapses are pivotal in neuron communication during distinct brain development ages and are strictly implicated in several processes, such as neuronal plasticity and learning processes [15,16].

The term “synaptopathy” refers to neurological disorders related to synapse dysfunction, mainly affecting synaptic transmission and/or plasticity [17,18,19].

Several genetic studies have recently identified different variants in pre-synaptic genes involved in Ca^2+^ related neurotransmitter releases such as *SYT1*, *VAMP2*, or *SNAP25* [20], and genes encoding key proteins involved in synaptic homeostasis such as neurexins (NRXNs) and neuroligins (NLGNs), synaptic adhesion proteins located on the pre- and post-synaptic membrane, respectively [21,22,23,24,25,26]; SHANK proteins involved in scaffolding excitatory glutamatergic synapses that bind NLGNs-NRXNs and NMDAR complexes at the postsynaptic density PSD [27,28,29]; synapsins (pre-synaptic proteins regulating neurotransmitter release) [30,31]; GABRs (GABA receptor, mediating inhibitory neurotransmission) [32,33,34]; NMDARs and AMPARs (for ionotropic glutamate transmission) receptor subunits (Table 1) [35,36,37,38].

We dissected the main emerging genetic synaptopathy associated with pediatric NDDs and epilepsy, highlighting recently identified molecular mechanisms, detailing, where present, the genotype–phenotype correlations.

## 2. Pre-Synaptic Genes

### 2.1. VAMP2

**Figure 1:**
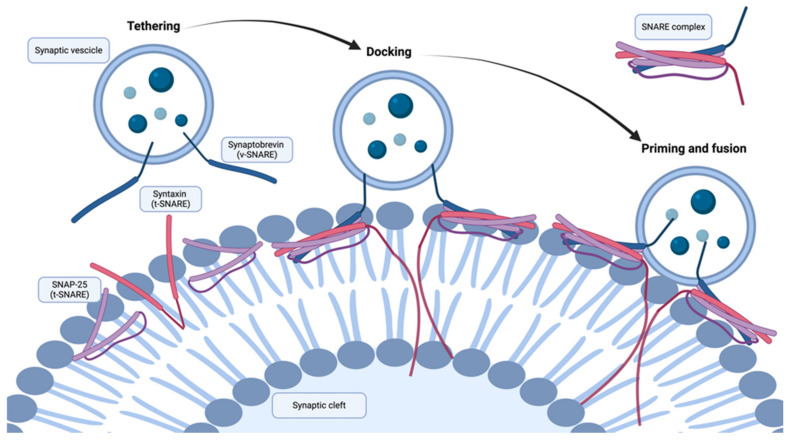
Steps in synaptic vesicle fusion and critical proteins involved (realized with BioRender.com, accessed on 10 April 2024). The exocytosis of synaptic vesicles includes different stages, such as tethering, docking, priming, and fusion. In the docking step, SNARE proteins interact with each other via SNARE motifs. The matching progresses from the N-terminus of the SNARE proteins to the C-terminal transmembrane regions, where the membranes interact. The resulting trans-SNARE complex firmly pulls the two membranes together (that of the synaptic vesicle and that of the plasma membrane of the nervous cell), providing the energy to fuse the lipid bilayers.

*VAMP2* (NM_014232) encodes a presynaptic protein, the vesicular v-SNARE protein VAMP2 (or synaptobrevin-2), involved in Ca^2+^-related neurotransmitter release. SNARE proteins mediate the attachment and fusion of synaptic vesicles at the plasma membrane of the axon during the neurotransmitter release process. Among SNARE proteins, *VAMP2* is involved in vesicular exocytosis and neurotransmitter release at the presynaptic level. It is located on the vesicle membrane (v-SNARE), and it interacts with target t-SNARE proteins syntaxin1A (STX1A) and synaptosomal-associated protein 25 Kd (SNAP25) located on the plasma membrane. When the v-SNARE interacts with the t-SNAREs, the helical domains of synaptobrevin wrap around the helical domains of the t-SNAREs to form a stable bundle. The resulting trans-SNARE complex firmly pulls the two membranes together (that of the synaptic vesicle and that of the plasma membrane of the nervous cell), providing the energy to fuse the lipid bilayers [132,133,134] (Figure 1).

This process is critical in maintaining proper neuronal communication, and any disruption in the VAMP2 protein or the SNARE complex can result in a variety of neurological impairments, including seizures. Seizures occur when there is abnormal, excessive neuronal activity in the brain, often linked to neurotransmitter release or synaptic transmission dysfunction. In the case of *VAMP2*-related dysfunction, improper synaptic vesicle fusion and impaired neurotransmitter release can lead to dysregulation of neuronal circuits, contributing to epileptic events.

*VAMP2* intragenic de novo variants were primarily identified in 2019 by Salpietro et al. (and confirmed by Sanger sequencing) in 5 individuals with neurodevelopmental impairment with variable neurological features [20]. More specifically, three de novo non-synonymous variants in *VAMP2* were described [p.Ser75Pro (c.223T>C)], [p.Phe77Ser (c.230T>C)], [p.Glu78Ala (c.233A>C)]; a de novo single amino acid deletion at position 43 [p.Val43del (c.128_130delTGG)] and a de novo single-amino-acid deletion at position 45 [p.Ile45del (c.135_137delCAT)]. The five children affected presented axial hypotonia since birth, ID, ASD, Rett-like features, speech impairment, and clinical or electrographic seizures, while three individuals had choreic movements [20]. Accordingly, in 2020, Simmons et al. [40] described five unrelated patients with de novo pathogenic variants in *VAMP2,* with global developmental delay, autistic features, behavioral disorders, and seizures. All patients carried de novo heterozygous variants. Interestingly, the missense variants of two patients’ Gly73Trp and Arg56Leu, associated with a more severe epileptic phenotype in vivo, exerted a dominant-negative effect on action potential (AP)-triggered SV fusion and neurotransmitter release in vitro. This highlights the significant impact of *VAMP2* dysfunction on synaptic transmission, which can lead to more severe forms of epilepsy characterized by frequent and often drug-resistant seizures.

Furthermore, Sunaga et al. [39] reported a heterozygous missense variant in *VAMP2* (c.199G>C, p.Ala67Pro) in an 8-year-old male with a severe neurological phenotype involving hypotonia and no visual pursuit, severe intellectual and motor disability (non-verbal, absence of purposeful hand movements, and a bedridden state), hyperkinetic movement, epilepsy and marked EEG abnormality. Impairment in vesicle fusion might be one potential pathogenetic mechanism related to these disorders.

Previously, other variants in genes encoding presynaptic proteins involved in Ca2+-regulated neurotransmitter release were described, unraveling this emerging spectrum of synaptopathies causing neurodevelopmental disorders and highlighting the key role of these genes in human brain development and function and its disruption as a significant contributor to epilepsy. In the context of *VAMP2* pathogenic variants, impaired vesicle fusion, and neurotransmitter release are central to the pathophysiology of epilepsy in these patients, leading to a cascade of abnormal neuronal activity that manifests as seizures.

### 2.2. SNAP25

SNAP25 is a t-SNARE protein with a key role in Ca^2+^-triggered exocytosis of synaptic vesicles: v-SNARE protein assembles with t-SNAREs (SNAP25 and syntaxin), mediating the fusion of synaptic vesicles with the presynaptic plasma membrane and allowing activity-dependent neurotransmitter release [135,136]. Seven *SNAP25* variants (NM_003081.4) have been reported to date in eight patients presenting with seizures, intellectual disability, speech delay, cerebellar ataxia, and muscle weakness. In 2013, Rohena et al. described a de novo variant [p.Val48Phe (c.142G>T)] in a girl [44]. In 2014, Schen et al. used WES to describe a *SNAP25B* de novo pathogenic variant in one affected child [p.Ile67Asn (c.200T.A)], resulting in an inhibition of synaptic vesicle exocytosis [43]. Moreover, in 2018, a novel missense *SNAP25* variant [p.Arg59Pro (c.176G>C)] inherited from the mosaic father was described by Fukuda et al. in two affected siblings (a female and a male) showing seizures and motor clumsiness; the female also presented delayed speech, cerebellar ataxia, hypotonia, learning disabilities, and ADHD [42]. Interestingly, Klöckner et al. [41] described 23 individuals with 19 de novo variants in *SNAP25* (15 different missense variants, four loss of function variants, two nonsense, and two splice donor variants). All individuals had a variable degree of ID and motor delay. Seizures were reported in 17 individuals who experienced multiple seizure types, such as epileptic spasms and generalized and focal seizures. Additionally, ataxia, dystonia, brain volume loss, muscular hypotonia, and spasticity were reported as recurrent clinical symptoms. All missense variants were located in the coiled-coil homology domains 1 and 2 of SNAP25, suggesting that they could represent potential hotspots.

### 2.3. SYT1

Synaptotagmin-1 (SYT1) is a calcium-binding synaptic vesicle protein. Synaptotagmins serve as calcium sensors that trigger neurotransmitter release, modulate vesicle exocytosis and endocytosis, and have a regulatory role in the trafficking of synaptic vesicles [137].

Baker et al. in 2015 described a heterozygous de novo non-synonymous *SYT1* variant (NM_005639.3) with an isoleucine-to-threonine substitution at position 368 (I368T) [46]. In 2018, Baker et al. [45] described other five de novo *SYT1* variants in a case series of 11 patients (M303K c.908T>A; D304G c.911A>G; I368Tc. 1103T>C; D366E c.1098C>A; D366E c.1098C>G; N371K c.1113C>G). These known *SYT1* variants may disrupt the protein’s calcium-sensing role and synaptic vesicle regulation, significantly impacting neurotransmitter release and synaptic plasticity [45,46,137].

Patients carrying *SYT1* pathogenic variants exhibit phenotypes characterized by hypotonia, developmental delay, congenital ophthalmic abnormalities, hyperkinetic movement disorders, and complex motor stereotypies. MRI is usually unremarkable, and EEGs are often characterized by typical low-frequency oscillation bursts [45,46].

### 2.4. UNC13A

*UNC13A* gene (NM_001080421.2) encodes for a protein with a role in vesicle maturation during exocytosis, allowing synaptic vesicle fusion. This fusion process is essential for proper synaptic transmission, and genetic deletion of *UNC13A* results in a complete block of synaptic communication [138]. In 2017, Lipstein et al. described a de novo variant in *UNC13A* (also known as Munc13-1) [p.Pro814Leu (c.2441C>T)] in one patient presenting with dyskinetic movement disorder, developmental delay, and ASD [47].

The role of *UNC13A* in epilepsy is closely linked to its involvement in synaptic transmission. Disruption in synaptic vesicle fusion can lead to abnormal neurotransmitter release, which in turn causes hyperexcitable neuronal circuits. Such dysregulation is a key mechanism underlying the development of epileptic seizures. Seizures are characterized by sudden bursts of abnormal electrical activity in the brain, often resulting from imbalances between excitatory and inhibitory signals. The gain-of-function variant in *UNC13A* identified by Lipstein et al. was shown to increase the fusion propensity of synaptic vesicles, leading to increased synaptic release probability, which could potentially create an environment favorable to seizure activity.

Using autaptic hippocampal neuronal cultures, the authors also demonstrated that the Pro-Leu exchange in *UNC13A* caused a gain of function of the Munc13-1 protein, resulting in increased fusion propensity of synaptic vesicles, leading to an increased synaptic release probability [47]. This increase in neurotransmitter release heightens the likelihood of excitatory imbalances at the synapse, a hallmark of epileptogenic activity. Therefore, while the variant was primarily associated with dyskinetic movement disorder, developmental delay, and ASD, it is plausible that the same synaptic dysfunction could contribute to epileptic activity, especially given that excessive neurotransmitter release as a factor in seizure generation.

### 2.5. STXBP1 (MUNC18-1)

*STXBP1* encodes a syntaxin-binding protein 1 (also known as MUNC18-1), mainly expressed in the brain, where it is critically involved in both vesicle trafficking and presynaptic neurotransmitter release. STXBP1 interacts with VAMP2 (v-SNARE located on the vesicle membrane), syntaxin-1, and SNAP25 (t-SNARE proteins located on the plasma membrane), modulating the vesicular fusion. The *STXBP1* (NM_003165.3) is made up of 20 exons and is located on chromosome 9q34.11 [139].

In 2016, Stamberger et al. described new *STXBP1* variants and reviewed previously reported genotype–phenotype correlations in a total of 147 patients [53]. *STXBP1* is one of the most frequent gene variants occurring in epilepsy associated with encephalopathy. Clinically, the cohort of patients described presented with ID, seizures, autistic features, hypotonia, ataxia, dyskinesia, and dystonia. Most of them had early-onset infantile epileptic encephalopathy (EIEE), or infantile epileptic spasm syndrome (IESS). The most frequent seizure types reported were epileptic spasms and tonic seizures. EEG may show focal or multifocal epileptic activity, burst-suppression, or hypsarrhythmia, and MRI may be normal or revealing cortical atrophy [53]. Abramov et al. in 2020 [52] described 282 patients with 237 variants in *STXBP1*, reported until then. The majority of these patients had epilepsy (85%), while all had ID. Interestingly, nonsense variants were associated with EIEE more than any other group, and fewer non-epileptic cases were observed in intragenic deletions/duplications. Moreover, missense variants were associated with variable phenotypes, suggesting that different missense variants might be associated with distinct clinical manifestations. Naseer et al. [49] described two 4-year-old females with heterozygous stop-gain and missense variants in the exon 5 of the *STXBP1* gene, leading to developmental and epileptic encephalopathy. Furthermore, Yang et al. [51] reported two children with epileptic encephalopathies with variable degrees of ID. Pathogenic deletion (of exons 13-20 and 3′ downstream of *STXBP1*) and nonsense variant [c.1663G>T (p.Glu555X) in exon 18 of *STXBP1*] were detected from the two patients, respectively. The RNA expression analysis revealed that both haploinsufficiency and truncation of STXBP1 protein (either with a dominant negative effect) might be causative mechanisms concerning the phenotypes observed. Wang et al. [48] reported a child with refractory epilepsy and neurodevelopmental delay carrying a heterozygous variant in the *STXBP1* gene (c.963 + 2T>C). Interestingly, he displayed startle-like responses that occasionally occurred at night, an uncommon *STXBP1*-related feature. Moreover, Takeda et al. [50] reported a female child with a pathogenic variant in the *STXBP1* gene, with developmental and epileptic encephalopathy (IESS), carrying an *STXBP1* gene variant (c.875G > A: p.Arg292His).

### 2.6. NRXN

NRXN is a heterogeneous group of presynaptic proteins encoded by different genes, such as *NRXN1* (NM_001330078.2), *NRXN2* (NM_015080.4), and *NRXN3* (NM_001330195.2). These proteins are presynaptic effectors that, by binding NLGNs on the postsynaptic membrane, lead to neurotransmitter release [140]. Exonic *NRXN1* deletions have been associated with neurodevelopmental delay, ASD, ID, schizophrenia, and Pitt-Hopkins syndrome [54,55,56,57,58,59,60]. Rare exonic microdeletions in the *NRXN3* locus 14q24.3–31.1 have been related to ASD, such as a deletion overlapping exon 1 of *NRXN3* alpha and a deletion overlapping multiple exons of the alpha and beta *NRXN3* isoforms, leading to a more important neurological involvement [22]. Moreover, in two siblings presenting with ASD and infantile spasms, *NRXN1* exon 2–5 homozygous deletion (chr2:51149007-51255411; 106.404 kb) has been detected [61]. A causative genotype–phenotype correlation has not been definitely established; however, homozygous *NRXN1* exonic deletions seem to be related to a variable clinical phenotype comprising ASD, severe ID, and epileptic encephalopathy [61,62]. Epilepsy is an important phenotype in individuals with *NRXN* (neurexin) gene variants. Particularly in the context of exonic deletions in *NRXN1*, patients presenting with developmental and epileptic encephalopathies, mainly including infantile epileptic spasms syndrome, with early onset epileptic spasms, developmental delays, and progressive neurocognitive impairment.

The presence of epilepsy, particularly featuring developmental and epileptic encephalopathy, alongside other neurodevelopmental symptoms, underscores the significance of neurexins in maintaining neural circuit stability and suggests a critical need for further research into their role in epileptic mechanisms.

### 2.7. CPLX1

The complexin-1 protein (CPLX1) is an important component of the complexin family of proteins. This presynaptic protein is encoded by the *CPLX1* gene (NM_006651.4) and has the function of interacting with the SNARE complex [141]. The two different isoforms, *CPLX1* and *CPLX2*, are mostly located within the central nervous system, triggering calcium release and vesicle fusion [63,64]. A few individuals with neurodevelopmental delay due to homozygous pathogenic *CLPX1* variants have been described, probably due to loss-of-function mechanisms [65,66]. Importantly, these *CPLX1* variants have also been associated with epileptic seizures and epilepsy. Given the crucial role of *CPLX1* in the regulation of synaptic vesicle fusion, its disruption can result in synaptic dysfunction, leading to neuronal hyperexcitability, a key factor in the generation of epileptic activity. Reported variants in *CPLX1* have predominantly been homozygous, which may indicate that complete loss of functional *CPLX1* protein is necessary to produce the neurodevelopmental and epileptic phenotypes observed. The exact variants have been associated with symptoms, including severe developmental delay, hypotonia, intellectual disability, and epileptic seizures [65,66]. While the focus has been on neurodevelopmental delay and epilepsy, other potential symptoms could include motor impairments and cognitive deficits, as synaptic function in *CPLX1*-related pathways is critical to a wide range of neuronal processes. Furthermore, given its role across inhibitory and excitatory synapses, *CPLX1* dysfunction could theoretically impact a wide range of other neuropsychiatric disorders, though research is still early in exploring these connections.

### 2.8. TBC1D24

*TBC1D24* (NM_001199107.2) is an additional critical gene for neuronal migration, synaptogenesis, and neurodevelopment through its presynaptic protein product TBC1D24 [67,68,141]. It plays an important role in modulating vesicle trafficking by regulating the GTPases Rab35 and Arf6 [18]. Various reports documented severe neurological involvement in patients with either homozygous or compound heterozygous pathogenic *TBC1D24* variants, probably resulting in an impaired protein and characterized by seizures, sensorineural hearing loss, cerebellar abnormalities, neurodegeneration, moderate to severe epileptic encephalopathy, and movement abnormalities [69,70,71,72,73,74,75,76,77,78,79].

Epileptic seizures are among the most prominent features associated with *TBC1D24* pathogenic variants. These patients often experience early-onset seizures, including infantile spasms, focal seizures, and, in some cases, generalized tonic-clonic seizures. The epileptic encephalopathy observed in individuals with *TBC1D24* variants is characterized by frequent, refractory seizures, which can contribute to severe cognitive decline and developmental regression. The epileptic activity in these patients often begins at a very young age and can be resistant to standard antiseizure medications, making seizure management particularly challenging.

The pathophysiology of epilepsy in *TBC1D24*-related disorders is likely tied to the gene’s role in synaptic vesicle trafficking and presynaptic neurotransmitter release. Disruption of the normal function of *TBC1D24* impairs the vesicle fusion process, leading to synaptic dysfunction and neuronal hyperexcitability. This imbalance in synaptic activity can result in synchronized neuronal discharges, which is the hallmark of epileptic seizures. Moreover, the dysregulation of Rab35 and Arf6 GTPases, modulated by TBC1D24, further impacts synaptic vesicle recycling, exacerbating the epileptic phenotype.

Patients with *TBC1D24* pathogenic variants often present with a combination of epileptic encephalopathy and neurodegenerative features, including cerebellar atrophy, which contributes to motor deficits such as ataxia and dystonia. In many cases, epilepsy manifests as a severe, progressive disorder where seizures increase in frequency and intensity over time, further impairing cognitive and motor functions.

### 2.9. DNM1

*DNM1* (NM_004408.4) encodes a key GTPase protein, mostly expressed in neurons and known as dynamin (DMN). This protein exists in three main isoforms: the first one is the most important, being involved in endocytosis, vesicle trafficking, and synaptogenesis [142]. Notably, a wide range of clinical phenotypes, ranging from ASD, cognitive impairment, and various types of epilepsy and seizures, have been related to de novo heterozygous missense or nonsense *DNM1* pathogenic variants, probably resulting in a disrupted protein [18].

Epileptic seizures in individuals with *DNM1* pathogenic variants often manifest in the context of a developmental and epileptic encephalopathy, characterized by frequent and severe seizures that can lead to developmental delays and cognitive decline. Various seizure types may be present, including focal seizures, generalized tonic-clonic seizures, and myoclonic seizures, reflecting the broad spectrum of epilepsy phenotypes associated with *DNM1* variants. In many cases, these seizures are resistant to standard antiseizure treatments, making seizure management particularly challenging.

Epileptic seizures in patients with *DNM1* variants are thought to be caused by the impairment in vesicle trafficking and endocytosis, which are essential for maintaining synaptic homeostasis. The failure to efficiently recycle synaptic vesicles results in an accumulation of neurotransmitters at the synapse, which can lead to hyperactive signaling and excessive neuronal firing—the core mechanism behind seizure activity. This disruption in the synaptic vesicle cycle causes neuronal circuits to become dysregulated, increasing the likelihood of synchronized neuronal discharges, which are characteristic of epileptic events.

In addition to seizures, individuals with *DNM1* variants often exhibit a range of neurodevelopmental abnormalities, including ID, ASD-like behaviors, and movement disorders, further complicating the clinical management of these patients. The combination of epileptic encephalopathy and cognitive impairment often leads to a progressive worsening of neurological function, as frequent seizures exacerbate the underlying neurodevelopmental deficits.

To date, 33 individuals with de novo heterozygous variants in *DNM1* have been described [80,81,82,83,84,85,86,87,88,89,90]. They presented a phenotype including severe developmental delay, ID, Lennox–Gastaut syndrome, infantile spasms and other types of seizures, and autistic traits. Pathogenic variants were both missense and nonsense, and they affected predominantly the GTPase or the middle domain of the protein, with a predicted loss of function of the protein [18].

### 2.10. PRRT2

The proline-rich transmembrane protein 2 (PRRT2) is a synaptic protein formed by two C-terminal transmembrane domains playing a critical role in its functioning and an N-terminal proline-rich extracellular domain. PRRT2 is mainly expressed at a presynaptic level in glutamatergic cerebral, basal ganglia, and cerebellar neurons [91,92]. While its precise mechanism of action is still not fully understood, it is thought to be involved in regulating neuron excitability through calcium-mediated neurotransmitter release, interacting with other components of the synaptic space, such as VAMP2, SYT 1-2, and SNAP-25 [93]. In addition, PRRT2 regulates neuronal migration during the early stages of nervous system development and synaptogenesis [18]. Pathogenic variants in *PRRT2* (NM_145239.3), including homozygous or compound heterozygous variants, are linked to gene haploinsufficiency and a wide range of phenotypes. *PRRT2* variants often result in loss of function, leading to insufficient levels of functional PRRT2 protein, which is characteristic of haploinsufficiency. These phenotypes include benign familial infantile epilepsy (BFIE), hemiplegic migraine, neurodevelopmental delay, cognitive impairment, and various psychiatric symptoms [18,93,94]. One of the most common presentations of *PRRT2* variants is BFIE, characterized by seizures that typically start in the first year of life. These seizures are often focal in nature and generally self-limited or well-controlled with antiseizure medications. However, in some cases, individuals with *PRRT2*-related epilepsy may develop more complex forms, where seizures become more frequent and harder to manage. The seizures associated with *PRRT2* variants are thought to arise from dysregulation of presynaptic calcium-mediated neurotransmitter release, leading to neuronal hyperexcitability, which is the key factor in generating epileptic activity.

The involvement of *PRRT2* in glutamatergic synaptic transmission is critical for maintaining the balance between excitatory and inhibitory signaling. When this balance is disrupted, as seen in *PRRT2* haploinsufficiency, the result is often abnormal neuronal firing and synchronized discharges, which underlie epileptic seizures. The precise interaction of PRRT2 with synaptic proteins like VAMP2, SYT1-2, and SNAP-25 further highlights its role in modulating synaptic vesicle release and calcium dynamics, both of which are essential for preventing excessive neuronal excitability.

Additionally, patients with *PRRT2* variants may exhibit a broader spectrum of non-epileptic phenotypes, including paroxysmal dyskinesias and migraine. This suggests that *PRRT2*-related disorders may involve a shared pathogenic mechanism that links seizures, movement disorders, and migraines to synaptic dysfunction. The presence of neurodevelopmental delays and cognitive impairments in many patients with *PRRT2* variants underscores the importance of early detection and management of epileptic seizures to prevent further neurological deterioration.

## 3. Glutamatergic Synaptic Transmission

**Figure 2:**
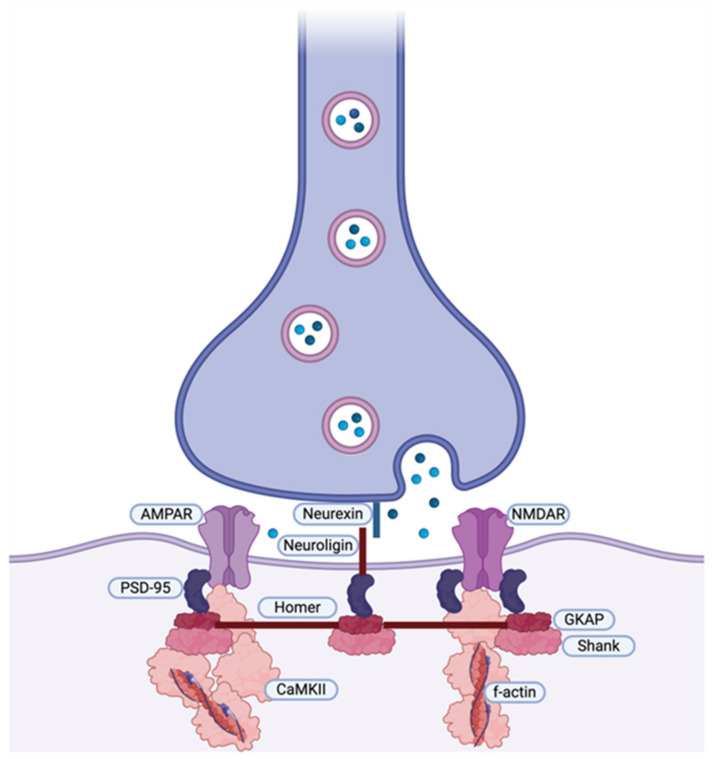
Organization of AMPA and NMDA receptors at the postsynaptic density (realized with BioRender.com, accessed on 10 April 2024).

Glutamatergic excitatory synaptic transmission in the central nervous system is mediated by three major subtypes of glutamate receptors: AMPA, NMDA, and kainate. Most postsynaptic excitatory neurons express both AMPA and NMDA receptors. They are tetrameric proteins with four subunits arranged around a central pore [143]. At excitatory synapses, the membrane of the postsynaptic cell is organized as a complex of molecules: a large number of regulatory proteins (including neuroligins, TARP, PSD-95, Shank, Homer, and others) form the postsynaptic density and help to organize the postsynaptic structure, localizing the postsynaptic receptors (NMDA and AMPA) [140,144] (Figure 2).

The role of glutamatergic transmission in epilepsy is critical, as dysregulation in glutamate receptor function can lead to hyperexcitability of neuronal networks, a key factor in the generation of seizures. Excessive activation of AMPA and NMDA receptors in the post-synaptic neurons can result in prolonged excitatory post-synaptic potentials, causing neuronal depolarization and increasing the likelihood of seizure activity. This is particularly relevant in epileptic disorders where an imbalance between excitatory and inhibitory neurotransmission contributes to synchronized neuronal firing, characteristic of epileptic seizures.

Pathogenic variants have been described in all these reported components in association with neurodevelopmental disorders, highlighting the importance of glutamatergic synaptic transmission in human synaptic plasticity and neurodevelopment [145,146].

The overactivation of glutamate receptors in excitatory synapses has been linked to refractory epilepsy, where seizures are frequent and resistant to standard antiepileptic treatments. This dysregulation in glutamate-mediated synaptic transmission not only contributes to the initiation of seizures but also plays a role in the progressive nature of certain epileptic syndromes, where repeated seizures lead to further synaptic plasticity alterations and network reorganization, exacerbating the condition over time.

Glutamatergic excitatory synaptic transmission in the central nervous system is mediated by the ionotropic glutamate receptors (iGluRs) comprising AMPA- and NMDA- and kainate-type receptors, and the metabotropic glutamate receptors (mGluRs). A large number of regulatory proteins (neuroligins, TARP, PSD-95, Shank, Homer, and others) form the postsynaptic density and help to organize the postsynaptic structure and the postsynaptic receptors (NMDA and AMPA). The ionotropic receptors are spatially and functionally segregated from the metabotropic receptors. AMPA and NMDA receptors are highly enriched in the core of the postsynaptic density.

### 3.1. AMPA Receptor: GLUA2, GLUA1, GLUA3, and GLUA4

AMPA receptors (AMPARs) are permeable to Na^+^ and K^+^. The AMPA receptor subunits are encoded by four different genes (GluA1-2-3-4), and most AMPA receptors are heterodimers made up of two types of GluA subunits. The most frequent combination is Glua1/Glua2.50. GluA2 subunits, encoded by *GRIA2*, have a key role in regulating AMPAR Ca^2+^ permeation and voltage rectification, reducing the channel conductance and making the hetero-multimeric AMPAR impermeable to Ca^2+^ [147,148].

In 2019, Salpietro et al. described 28 unrelated individuals carrying *GRIA2* (NM_001083619.3) de novo intragenic variants and the majority of the variants revealed an AMPAR loss of function [101]. The phenotype of the affected children was characterized by ID, ASD, Rett-like features, speech impairments, seizures, ataxia, dyspraxia, and abnormal sleep rhythm. EEG showed epileptic activity, and some MRI scans showed cerebellar atrophy. Previously, two other reports described two patients with a *GRIA2* variant and a neurodevelopmental disorder. Hackmann et al., in 2013 [103], was the first to report a *GRIA2* pathogenic variant in a patient presenting with ID, speech delay, gait abnormalities, and ADHD; one other *GRIA2* variant was described in an affected child presenting with ID recruited in the Deciphering Developmental Disorders (DDDs) study [102]. The other GluA genes associated with NDDs have also described rare variants. In 2021, Zhou et al. [100] described a 3-year-old girl carrying a de novo missense variant, c.1934T>G (p.Leu645Arg), in the *GRIA2* gene. The phenotype included delayed language and stereotyped, compulsive behavior. Furthermore, Alkelai et al. reported in 2021 [99] a de novo stop variant in the *GRIA2* gene (c.1522 G>T (p.Glu508Ter) in a 10-year-old female with behavioral abnormalities, obsessive-compulsive disorder (OCD), ADHD, aggressive behavior, schizophrenia, and early life motor and language delay. In 2022, Latsko et al. [98] described a 4-year-old male with epileptic encephalopathy characterized by seizures, ASD, and global developmental delay. WES revealed a de novo missense variant in *GRIA2* (c.1589A>T; p.Lys530Met) functionally associated with the AMPA receptor’s key ligand binding domain. Interestingly, Cai et al. [97] documented a de novo *GRIA2* variant (c.2308G>A, p.Ala770Thr) in a four-year-old girl with behavior regression and psychiatric symptoms such as stereotyped behaviors, language regression, social interactions, and visual hallucinations. This variant was predicted to result in instability of the protein structure.

In 2017, Geisheker et al. described one recurrent site substitution (p.A636T) in the glutamate receptor subunit, *GRIA1* (NM_000827.4), in five patients presenting with a similar phenotype [96]. The variant is localized in a highly conserved region in the M3 transmembrane domain of glutamate receptors, playing a critical role in channel gating. Affected individuals presented with ID, ASD, language delay, and normal MRI. In 2022, Ismail et al. [95] reported seven affected patients, six with missense heterozygous variants and one homozygous nonsense variant in *GRIA1*. The associated phenotype included ID, speech and language delay, sleep difficulties, abnormal EEG with or without seizures, normal brain imaging, and endocrine abnormalities. Interestingly, the autosomal recessive inheritance pattern was associated with a more severe phenotype compared to the autosomal dominant one. It was characterized by early-onset seizures and profound speech and language delay, suggesting that the homozygous nonsense variants resulted in a nonfunctioning *GRIA1* gene.

In 2017, Davies et al. [110] described two male children with a severe developmental disorder and altered sleep-wake cycle, characterized by up to 106 h awake and 48 h asleep. In the X-linked *GRIA3* gene (NM_007325.5), a novel missense variant in a highly conserved transmembrane domain of the ion channel gate was identified in both siblings (c.Ala653Thr). The importance of the *GRIA3* gene in regulating the sleep-wake cycle was also validated using a gene-edited mouse. Four *GRIA3* missense variants (c.Gly833Arg, c.Met706Thr, c.Arg631Ser, and c.Arg450Gln) were previously identified in 2007 in patients with ID [149]. Melnikova et al. [106] described a female pediatric patient with developmental and epileptic encephalopathy carrying a de novo missense variant in *GRIA3* (c.2359G>A; p.Glu787Lys). Interestingly, functional studies showed a significantly lower iGluR3 expression in the patient’s fibroblasts than in controls and different responses to glutamate treatment. Sun et al. [107], reported a de novo pathogenic missense variant in *GRIA3* (c.1979G>C; p. R660T) identified in a 1-year-old female patient with severe epilepsy and global developmental delay. Notably, functional studies revealed that the gain of function variant in *GRIA3* might cause epileptic encephalopathy and global developmental delay in a female subject by enhancing synaptic transmission. Moreover, Rinaldi et al. [105] reported a novel *GRIA3* variant, c.2360A>G p.(Glu787Gly) in an 11-year-old-male, with developmental delay evolving in severe ID, cerebellar signs due to vermian hypoplasia, clonic/myoclonic seizures even configuring myoclonic status epilepticus, short stature, low weight, relative macrocephaly, and facial dysmorphisms. Okano et al. [104] described a 13-year-old female with developmental and epileptic encephalopathy who carried a de novo heterozygous variant in *GRIA3* (c.1982T>C: p.Met661Thr). She was bedridden with hypertonia and severe psychomotor delay. Trivisano et al. [108] reported a 26-month-old boy with developmental delay, early-onset refractory myoclonic epilepsy, and non-convulsive refractory status epilepticus, carrying a hemizygous missense variant (c.2359G>A, p.Glu787Lys) in *GRIA3*. Moreover, Bai et al. [109] described a 13-year-old male with developmental delay and mild EEG discharges carrying a hemizygous variant in *GRIA3* (c.64C>T, p.Leu22Phe).

Martin et al. in 2017 reported five de novo pathogenic variants in five unrelated individuals with a neurodevelopmental disorder [112] [c.1915A>T (p.Thr639Ser), c.1921A>G (p.Asn641Asp), c.1928C>G (p.Ala643Gly), c.1931C>T (p.Ala644Val), and c.2090G>C (p.Arg697Pro)]. The phenotype was characterized by ID, speech difficulties, gait abnormalities, and seizures [112]. In 2022, Wang et al. [111] described a 9-month-old girl with severe developmental delay, limb hypertonia, focal seizures, retinal hypoplasia, chorioretinal hyperpigmentation, tricuspid defect, carrying a de novo heterozygous variant in *GRIA4* (NM_000829.4) (c.1918G>T, p.Ala640Ser).

### 3.2. NMDA Receptors

Some important postsynaptic ionotropic receptors for excitatory glutamatergic transmission are the N-methyl-D-aspartate (NMDA) receptors (NMDARs). Such receptors are permeable to ionized calcium, sodium, and potassium and are normally blocked by ionized magnesium when at resting potential. Structurally, NMDARs are hetero-tetramers with two NR1 and two NR2 (A, B, C, or D) and/or NR3 (A or B) subunits. The *GRIN2A* (NM_001134407.3) and *GRIN2B* (NM_000834.5) genes encode the GluN2A and GluN2B subunits activated by glutamate. Instead, the GluN1 subunit represents the binding site for glycine [150]. Notably, NMDARs are central structures for neurodevelopment and neural plasticity [151,152]. In 2011, a de novo *GRIN2B* pathogenic variant (c.2473T4G; p.L825V) was reported for the first time, and in the same paper, two additional de novo pathogenic *GRIN2A* variants were identified in two patients with sporadic schizophrenia. Three de novo *GRIN2B* (a nonsense, a slice, and a frameshift) variants were first described in 2012, associated with autism spectrum disorder [36]. In 2010, four de novo *GRIN2B* pathogenic variant abnormalities were identified (c.411+1G>A, c.2044C>T, c.2360-2A>G, and c.803_804delCA) and reported in four patients with neurodevelopmental delay, cognitive impairment, and behavioral changes [153]. Moreover, murine models have documented relevant associations between NMDAR impairment and ASD [154,155]. Specifically, a well-known murine model based on a constitutive reduction in NR1 subunit expression documented behavioral abnormalities, ASD, and reduced social interactions [154,155]. Also, recent studies focused on such pathways, such as a report published in 2022 with two patients with autism spectrum disorders, language delay, cognitive impairment, and focal epilepsy associated with previously reported heterozygous de novo *GRIN2A* pathogenic variants [156]. This rare clinical presentation has been related to the dysfunction of an aberrant GluN2A subunit and the interactions of tri-hetero-tetrameric 2GluN1/GluN2A-D/GluN3A-B subunits. In general, *GRIN2B* dysfunction has been mostly associated with cognitive delay, autism spectrum disorder, schizophrenia, and behavioral abnormalities, while *GRIN2A* impairment has been mainly related to a more specific phenotype with predominant epilepsy, ranging from the common benign childhood epilepsy with centrotemporal spikes to the rarer Landau–Kleffner syndrome, but also speech delay and intellectual disability. Lastly, 11 children with variable phenotypes due to *GRIN2B* pathogenic (mostly missense) variants have been recently described [157]. Such patients ranged from mild cognitive impairment to severe developmental delay and life-threatening epileptic encephalopathy. Nevertheless, the precise molecular genotype–phenotype correlation is still elusive. Clinical heterogeneity could depend on the type of genetic abnormality (i.e., the domain of GluN2A-B that is altered and the impact on protein function and structure), but it could also be related to environmental factors such as neurotoxins and a wide range of drugs [158].

## 4. Postsynaptic Genes

### 4.1. SHANK

The Shank proteins are a heterogeneous family of proteins encoded by similar genes located on different chromosomes. The main genes responsible for Shank protein production are *SHANK1*(NM_016148.5) (located on 19q13.33), *SHANK2* (NM_012309.5) (located on 11q13.2), and *SHANK3* (NM_001372044.2) (located on 22q13.3). These proteins play a critical role in organizing and assembling the large postsynaptic complex [135].

Shank proteins are essential for maintaining the structure and function of glutamatergic synapses, particularly in regulating the excitatory postsynaptic density where receptors like NMDA and AMPA are anchored. Dysfunction in these proteins can lead to synaptic dysregulation, which is a well-known factor in the development of epileptic seizures. *SHANK1*, *SHANK2*, and *SHANK3* variants have been associated with the development of ASDs, but they are also increasingly recognized as contributing to epilepsy, particularly through their role in synaptic excitability and plasticity.

In various murine models, mice with genetic dysfunction of the Shank proteins developed autistic behaviors, including repetitive actions and abnormal vocalizations, mirroring human symptoms [159,160,161,162,163,164,165]. In these models, mice with genetic dysfunction of the shank proteins developed autistic behaviors, especially repetitive actions (jumping, grooming) and vocalizations, similar to human changes [27]. Moreover, these models also displayed increased susceptibility to seizures, linking Shank protein dysfunction to both ASDs and epilepsy. The excitatory/inhibitory imbalance caused by the disruption of Shank proteins can lead to hyperexcitability in neural circuits, which is a hallmark of epileptic activity.

Among the clinical syndromes associated with Shank protein dysfunction, the 22q13.3 microdeletion syndrome (also known as Phelan-McDermin syndrome) is strongly associated with the onset of ASDs, neurodevelopmental delay, language impairment, and hypotonia. It has been found to be dependent on dysfunction in the *SHANK3* gene in over one thousand patients [113,114,115,116]. In addition to developmental and behavioral challenges, a significant number of patients with *SHANK3* variants also experience epileptic seizures, reflecting the critical role of *SHANK3* in maintaining the balance between excitatory and inhibitory signals in the brain. This imbalance can lead to epileptic encephalopathies, where seizures contribute to the deterioration of cognitive and motor functions over time.

Pathogenic variants in *SHANK1*, *SHANK2*, and *SHANK3* (including missense variants or microdeletions) have been related to non-syndromic forms of ASDs but also to epileptic disorders [28,117,118,119,120,121,122].

The severity of epilepsy in patients with Shank protein disruptions tends to correlate with the specific gene affected. For example, *SHANK3* variants are generally associated with more severe forms of epilepsy and cognitive impairment, while *SHANK1* variants are linked to milder neurodevelopmental and epileptic presentations [117].

A recently reported case described a 10-year-old patient with a de novo heterozygous *SHANK3* frameshift variant (c.1231delC; p.Arg411Val). This patient presented with severe ASD, neurodevelopmental delay, ADHD, language regression, and a history of seizures. These findings highlight the potential for *SHANK3* variants to contribute to epileptic syndromes in addition to their role in neurodevelopmental disorders [123].

### 4.2. NLGN

Neuroligins (NLGNs) are cell-adhesion proteins implicated in postsynaptic mechanisms due to their binding with their presynaptic counterpart cell-adhesion molecules, neurexins (NRXNs). NLGNs are key actors in synapse functioning, forming a trans-synaptic complex with NRXNs [140].

Such molecules are related to non-syndromic monogenic forms of ASD but also epilepsy. Proper interaction between NLGNs and NRXNs is critical for maintaining the synapse balance of excitatory and inhibitory signals. Any disruption in this process can lead to synaptic dysfunction, contributing to neuronal hyperexcitability, which is a key factor in the development of epileptic seizures.

Recently, frameshift variants in *NLGN4* (NM_181332.3) (such as the 1186insT variant) and missense variants in *NLGN3* (NM_181303.2) (such as c.1351C>T, p.Arg451Cys) have been associated with ASD in individuals with X-linked inherited forms of these disorders [124,125]. In addition to autistic features, individuals with these variants may exhibit seizures, linking NLGN dysfunction to epileptogenesis. A recent study described a European family of 13 males with either ASD or cognitive impairment due to a deletion in the fifth exon of *NLGN4*, further demonstrating the role of *NLGN* variants in both neurodevelopmental and epileptic phenotypes [125].

The synaptic dysfunction caused by *NLGN* variants affects excitatory synaptic transmission, which is highly dependent on the proper function of the NLGN-NRXN complex. In two families with multiple members exhibiting ASD and neurodevelopmental delay, missense variants in *NLGN3* (c.1789C>T, p.Arg597Trp, and c.1540C>T, p.Pro514Ser) were found [24]. These variants impair the proper expression of mature NLGN3 protein, disrupting the NRXN-NLGN interaction, which is crucial for normal excitatory synapse functioning. This dysfunction can result in neuronal hyperexcitability and increase the susceptibility to epileptic seizures.

The reduction in synaptic inhibition combined with enhanced excitatory transmission caused by NLGN and NRXN dysfunction is known to contribute to seizure generation. The resulting imbalance in neural circuits often leads to the development of epileptic encephalopathies, where frequent seizures can further impair cognitive and motor functions. In addition, the synaptic plasticity mediated by the NLGN-NRXN complex is also essential for maintaining normal cognitive processes. Therefore, variants in these genes not only predispose individuals to ASD but also increase the likelihood of developing epilepsy, particularly in the context of early-onset seizures and developmental delay.

### 4.3. DLG4

The postsynaptic density protein 95 (PSD-95), also known as disk large homolog 4 (DLG4), is encoded by the *DLG4* gene (NM_001321075.3) [19]. This protein plays a crucial role in the organization of the postsynaptic density components, which are pivotal structures for synaptic signaling, leading to the organization and development of excitatory glutamate-dependent synapses. *DLG4* comprises various subunits, including a SH3 domain, a GK domain, and three PDZ domains, all necessary for interacting with several receptors, ion channels, and other molecules often involved in congenital pediatric-onset cognitive impairment [19].

Disruption of *DLG4*, particularly through truncating variants, leads to non-functioning glutamatergic synapses, severely impairing excitatory synaptic transmission. This synaptic dysfunction can result in an imbalance between excitatory and inhibitory neurotransmission, a key factor in the development of epileptic seizures. Synaptopathies related to *DLG4* dysfunction are often characterized by neuronal hyperexcitability, which is closely linked to the onset of epileptic activity.

Several patients with pathogenic *DLG4* variants and neurological manifestations have been described [19]. Among these manifestations, epileptic seizures are particularly prominent. Seizures result from the disruption of glutamatergic signaling, where an imbalance in synaptic transmission leads to excessive neuronal firing, creating the conditions for seizure activity. Patients with *DLG4* variants typically experience early-onset epilepsy, often alongside cognitive impairment, ASD, and speech delays. In addition to seizures, other neurological features associated with *DLG4* variants include dystonia, tremor, hypotonia, and migraine [126]. The presence of these symptoms, in combination with epilepsy, highlights the role of PSD-95 in maintaining proper neuronal function and synaptic stability. Disruption of this critical scaffolding protein compromises the ability of glutamatergic synapses to regulate neural circuits, predisposing patients to seizure disorders.

The relationship between *DLG4* variants and epilepsy is particularly significant in the context of synaptopathies, where abnormal synaptic architecture and neurotransmission lead to refractory epilepsy in some cases. The altered postsynaptic density resulting from PSD-95 dysfunction interferes with the coordination of excitatory inputs, resulting in synchronized neuronal discharges that underlie epileptic episodes. This synaptic instability makes individuals with *DLG4* variants particularly vulnerable to epileptic encephalopathies, where ongoing seizures further compound neurodevelopmental delays and cognitive impairment.

### 4.4. KALRN

The Kalirin protein, a key synaptic regulator belonging to the wide family of Rho-guanosine nucleotide exchange factors (GEF), is encoded by the *KALRN* gene (NM_001388419.1) [127]. Various Kalirin isoforms exist that are produced through alternative splicing. Each isoform has its own different expression pattern; for example, Kalirin9 and Kalirin12 are expressed during embryonic neuronal development, whereas Kalirin7 has been found only in the adult cortex. This class of synaptic proteins shares many functions in the central nervous system, including regulating precocious neuronal growth, dendritic arborization, and glutamatergic receptor activity. Kalirin proteins exert their effects at the postsynaptic level through Rac- and RhoA-specific GEF activity, mediating cytoskeletal rearrangements that are crucial for synaptic plasticity. Pathogenic *KALRN* variants mostly have a loss-of-function or truncating significance, suggesting a haploinsufficiency molecular mechanism responsible for neurodevelopmental delay and cognitive impairment [102,127,131]. In addition to NDDs such as ASD, *KALRN* variants have also been implicated in epilepsy. Disruption of Kalirin function can lead to synaptic instability and neuronal hyperexcitability, both of which are key factors in the development of epileptic seizures. The role of Kalirin in synaptic regulation is particularly important for maintaining the balance between excitatory and inhibitory neurotransmission. Given that Kalirin influences the activity of glutamatergic receptors, its dysfunction can lead to an imbalance favoring excessive excitatory signaling, which is a hallmark of epilepsy. Hyperactive glutamatergic transmission can result in abnormal neuronal firing and the generation of seizures, particularly in individuals with *KALRN* variants.

A de novo pathogenic *KALRN* c.6070A>G; p.N2024D variant and an additional frameshift variant were documented in two different reports as being related to ASD [128,129]. Interestingly, *KALRN* loss-of-function variants have also been associated with schizophrenia, probably due to the impairment of both Rac-GEF and RhoA-GEF [38,130].

## 5. Conclusions

Neurodevelopmental disorders (NDDs) are a group of heterogeneous and complex conditions associated with significant phenotypic variability. Their pathogenesis is multifactorial, involving a combination of genetic, epigenetic, and environmental factors that interact to produce the wide spectrum of clinical manifestations observed. In the last decade, the advent of NGS technologies has shed new light on the molecular mechanisms underlying these multifaceted disorders, facilitating the description of genotype–phenotype correlations. Over the years, numerous studies have emphasized the crucial role of synapses in neurodevelopment, and several synaptopathies have been associated with NDDs. These disorders arise from synaptic dysfunction during different phases of brain development, leading to a complex neurological phenotype often coexisting with extra-neurological comorbidities and multisystemic involvement, such as gastrointestinal issues (feeding difficulties, gastroesophageal reflux, or constipation), hypotonia, failure to thrive, and growth and development restriction, leading to complex health challenges that require multidisciplinary management.

A key aspect of many NDDs is their frequent association with epilepsy. Defects in pre- and post-synaptic transmission can lead to neuronal hyperexcitability, a hallmark of epileptic activity. Synaptic impairment affects the delicate balance between excitatory and inhibitory neurotransmission, which is crucial for maintaining neuronal stability. Any disruption in this balance increases the likelihood of synchronized neuronal discharges leading to epileptic seizures.

Presynaptic synaptopathies (e.g., SNAREopathies, involving SNARE complex proteins) primarily affect the machinery responsible for neurotransmitter release, leading to deficits in synaptic vesicle docking, fusion, and release. Clinically, presynaptic dysfunction can result in a broader impact on neural circuits and is often associated with NDDs and epilepsy. In particular, SNAREopathies refer to disorders caused by variants in the SNARE complex proteins, which are essential for the fusion of synaptic vesicles with the presynaptic membrane and the subsequent release of neurotransmitters. Variants in genes encoding SNARE-related proteins, such as *VAMP2, STXBP1,* and *SNAP25*, have been implicated in both neurodevelopmental delay and severe developmental and epileptic encephalopathies. These variants disrupt the vesicle fusion process, impairing synaptic transmission and leading to neuronal hyperexcitability. Patients with SNAREopathies often exhibit early-onset seizures, which can be refractory to conventional antiseizure medications, further complicating their clinical management.

Postsynaptic synaptopathies (e.g., AMPA receptor-related synaptopathies) impact receptor trafficking, clustering, and signal transduction on the receiving side of the synapse. Such dysfunctions can lead to problems with synaptic plasticity, a process essential for learning and memory, and are often associated with cognitive impairment and neuropsychiatric symptoms. AMPA-related synaptopathies involve variants in genes encoding for AMPA receptor subunits, which are key players in excitatory synaptic transmission. Disruptions in AMPA receptor function led to increased excitatory signaling, contributing to the development of epileptic seizures. These receptors are critical for synaptic plasticity and the regulation of neuronal networks. Pathogenetic variants affecting AMPA receptor trafficking or function can result in uncontrolled excitatory neurotransmission, a primary mechanism behind seizure generation in many forms of epileptic encephalopathy. Patients with AMPA receptor dysfunction may experience a broad spectrum of epilepsy phenotypes, ranging from focal seizures to more generalized and severe forms of epilepsy.

The association between synaptic dysfunction and epilepsy underscores the importance of understanding the molecular underpinnings of these disorders. Many NDDs with epileptic manifestations involve variants in genes that regulate the formation, maintenance, and plasticity of synapses. Presynaptic proteins such as STXBP1 and TBC1D24 are essential for proper synaptic vesicle dynamics, and their disruption can lead to abnormal neurotransmitter release and epileptic seizures. Likewise, postsynaptic proteins like PSD-95, Shank, and Neuroligins, which play pivotal roles in organizing the postsynaptic density and ensuring effective receptor signaling, are frequently implicated in NDDs associated with seizures.

A prompt and accurate diagnosis of these conditions is crucial for optimizing patient care and improving patients’ and their families’ quality of life. Genetic testing should be routinely performed in all patients displaying NDDs associated with epilepsy or extra-neurological comorbidities, as identifying the underlying genetic cause can guide personalized treatment strategies. This is particularly important given that many synaptic disorders can present with developmental and epileptic encephalopathy, where early intervention can modify the clinical trajectory and improve outcomes.

Concurrently, functional animal model studies and clinical trials on patients with synaptopathies and associated epilepsy should be carried out to enhance our understanding of the pathogenesis of these disorders. By deepening our knowledge of the molecular mechanisms involved in pre- and post-synaptic transmission, we can develop targeted therapies to restore synaptic function and potentially modify the natural history of these complex conditions.

Further understanding of the specific pathways affected in presynaptic versus postsynaptic synaptopathies will be crucial in developing more precise and effective treatments.

As for many other monogenic diseases with epilepsy, the variability in phenotypic expression could arise from several factors, including the possibility that a single variant may lead to multiple phenotypes, reflecting these genes’ broad roles in neural development, synaptic function, and brain connectivity. In other cases, different variants within the same gene may result in distinct phenotypes. It is also possible that certain gene variants may predispose individuals to a range of symptoms, but the exact phenotype observed can vary depending on additional genetic modifiers, early developmental factors, or environmental interactions.

Phenotypes may also be impacted by epigenetic modifications, such as DNA methylation, histone modification, and non-coding RNA regulation, which have been implicated in synaptic development and plasticity, processes essential for normal neurodevelopment. These modifications can alter the expression of genes critical for synaptic function without changing the underlying DNA sequence, and disruptions in these processes are increasingly recognized in NDDs and epilepsy syndromes.

Given the genetic and clinical heterogeneity of NDDs and epilepsy, broad genetic testing (including NGS panels, CNV analysis, WES, and, when relevant, epigenetic testing) is recommended to capture the full range of possible pathogenic variants. Genetic testing results can significantly influence treatment decisions by identifying gene-specific therapeutic strategies, guiding medication choices, and potentially opening avenues for future gene or epigenetic therapies. As research advances, genetic insights will likely continue to play an increasingly central role in developing tailored, precision-based treatments for individuals with NDDs and epilepsy.

In conclusion, NDDs often involve a combination of synaptic dysfunction and epileptic activity, making it essential to approach these disorders with a comprehensive diagnostic and therapeutic strategy. By integrating genetic testing, functional studies, and personalized treatment approaches, we can improve the outcomes for individuals affected by these debilitating conditions and provide better support to their families.

## Figures and Tables

**Table 1:** Pre-synaptic, post-synaptic, and synaptic regulatory genes: phenotypes including the epilepsy details.

Gene	Phenotype	Epilepsy Details	References
*VAMP2* (NM_014232)	Axial hypotonia, ID, ASD, Rett-like features, speech impairments, epileptic seizures, chorea.	Severe early-onset epileptic seizures.	Sunaga et al., 2020 [39] Simmons et al., 2020 [40] Salpietro et al., 2019 [20]
*SNAP25* (NM_003081.4)	ID, speech delay, cerebellar ataxia, muscle weakness, epileptic seizures, DEE, dystonia, brain volume loss, spasticity.	DEE, with early-onset seizures and progressive loss of motor functions.	Klöckner et al., 2021 [41] Fukuda et al., 2018 [42] Shen et al., 2014 [43] Rohena et al., 2013 [44]
*SYT1* (NM_005639.3)	Hypotonia, congenital ophthalmic abnormalities, hyperkinesia, motor stereotypies, developmental delay.	Motor abnormalities and early-onset epilepsy with frequent myoclonic seizures.	Baker et al. (2018) [45] Baker et al. (2015) [46]
*UNC13A* (NM_001080421.2)	Dyskinetic movement disorder, developmental delay, ASD.	Rare early-onset epilepsy typically associated with motor delays.	Lipstein et al., 2017 [47]
*STXBP1* (NM_003165.3)	ID, epileptic seizures, autistic features, hypotonia, ataxia, DEE, developmental delay.	STXBP1 encephalopathy often features infantile spasms and severe, drug-resistant seizures, including tonic seizures.	Wang et al., 2023 [48] Naseer et al., 2022 [49] Takeda et al., 2022 [50] Yang et al., 2021 [51] Abramov et al., 2020 [52] Stamberger et al., 2018 [53]
*NRXN* (NRXN1 NM_001330078.2; NRXN2 NM_015080.4; NRXN3 NM_001330195.2)	ASD, language delay, seizures, ID.	Epilepsy ranging from focal seizures to developmental epileptic encephalopathy.	Vaags et al., 2012 [22] Pinto et al., 2010 [54] Ching et al., 2010 [55] Guilmatre et al., 2009 [56] Levy et al., 2011 [57] Sanders et al., 2011 [58] Zweier et al., 2009 [59] Südhof et al., 2008 [60] Aksu Uzunhan et al., 2022 [61] Imitola et al., 2014 [62]
*CPLX1* (NM_006651.4)	Developmental delay, motor dysfunction, seizures, ID.	Severe drug-resistant seizures, typically early-onset, and contribute to progressive neurodevelopmental decline.	Melland et al., 2021 [63] Trimbuch et al., 2016 [64] Redler et al., 2017 [65] Karaca et al., 2015 [66]
*TBC1D24* (NM_001199107.2)	Epileptic encephalopathy, neurodegeneration, movement disorders.	Severe epileptic encephalopathy with recurrent seizures resistant to treatment, often progressing with neurodegeneration.	Falace et al., 2014 [67] Kim Nguyen et al., 2020 [68] Lüthy et al., 2019 [69] Campeau et al., 2014 [70] Balestrini et al., 2016 [71] Salemi et al., 2020 [72] Fang et al., 2021 [73] Lozano et al., 2016 [74] Nakashima et al., 2019 [75] Banuelos et al., 2017 [76] Appavu et al., 2016 [77] Uzunhan et al., 2020 [78] Zhang et al., 2019 [79]
*DNM1* (NM_004408.4)	Severe developmental delay, ID, movement disorders, seizures.	DEE, drug-resistant epilepsy, frequently involving multiple seizure types.	Fung et al., 2017 [80] Lazzara et al., 2018 [81] Appenzeller et al., 2014 [82] Nakashima et al., 2016 [83] Li et al., 2019 [84] Deng et al., 2016 [85] Brereton et al., 2018 [86] Epi4K Consortium 2013 [87] Kolnikova et al., 2018 [88] Von Spiczak et al., 2017 [89] Mastrangelo et al., 2017 [90]
*PRRT2* (NM_145239.3)	Paroxysmal dyskinesia, hemiplegic migraine, epilepsy.	Benign familial infantile epilepsy, with episodes of paroxysmal movement and hemiplegic migraine, often seizure-free after childhood.	Valtorta et al., 2016 [91] Valente et al., 2016 [92] Fruscione et al., 2018 [93] Najmabadi et al. 2011 [94]
*GRIA1* (NM_000827.4)	ID, ASD, language delay, sleep disturbances, EEG abnormalities, endocrine abnormalities.	EEG abnormalities often associated with focal and generalized seizures.	Ismail et al., 2022 [95] Geisheker et al., 2017 [96]
*GRIA2* (NM_001083619.3)	Neurodevelopmental abnormalities, ID, ASD, Rett-like features, speech impairments, schizophrenia.	Focal seizures, myoclonic seizures, progression to epileptic encephalopathy.	Cai et al., 2022 [97] Latsko et al., 2022 [98] Alkelai et al., 2021 [99] Zhou et al., 2021 [100] Salpietro et al., 2019 [101] DDD Study, 2017 [102] Hackmann et al., 2013 [103]
*GRIA3* (NM_007325.5)	Neurodevelopmental delay, severe sleep-wake cycle alteration, DEE, myoclonic status epilepticus.	Severe epilepsy, myoclonic status epilepticus, and non-convulsive seizures.	Okano et al., 2023 [104] Rinaldi et al., 2022 [105] Melnikova et al., 2022 [106] Sun et al., 2021 [107] Trivisano et al., 2020 [108] Bai et al., 2019 [109] Davies et al., 2017 [110]
*GRIA4* (NM_000829.4)	ID, gait abnormalities, heart and ocular impairments, epileptic seizures.	Focal and tonic-clonic seizures, progression to epileptic encephalopathy.	Wang et al., 2022 [111] Martin et al., 2017 [112]
*SHANK* (SHANK1 (NM_016148.5; SHANK2 NM_012309.5; SHANK3 NM_001372044.2)	ASD, intellectual disability, hypotonia, speech impairment, seizures.	Epilepsy is frequent, often involving generalized tonic-clonic seizures and developmental delays.	Monteiro et al., 2016 [28] Molloy et al., 2023 [113] Wilson et al., 2008 [114] Phelan et al., 2011 [115] Dhar et al., 2010 [116] Leblond et al., 2014 [117] Boccuto et al., 2013 [118] Gauthier et al., 2009 [119] Gauthier et al., 2010 [120] Berkel et al., 2010 [121] Moessner et al., 2007 [122] Kanani et al., 2018 [123]
*NLGN* (NLGN4 NM_181332.3; NLGN3 NM_181303.2)	ASD, intellectual disability, language delay, seizures.	Focal and generalized seizures.	Quartier et al., 2019 [24] Paris Autism Research International Sibpair Study, 2003 [124] Laumonnier et al., 2004 [125]
*DLG4* (NM_001321075.3)	ID, epilepsy, ASD, cognitive impairment.	Severe drug-resistant seizures, with progressive developmental decline and epilepsy.	Rodríguez-Palmero et al., 2021 [19] Tümer et al., 1993 [126]
*KALRN* (NM_001388419.1)	Schizophrenia, ID, seizures, developmental delay.	Early-onset seizures typically focal and often progressing to encephalopathy.	Parnell et al., 2021 [127] Leblond et al., 2019 [128] Satterstrom et al., 2020 [129] Howrigan et al., 2020 [130] Makrythanasis et al., 2016 [131]

ASD = autism spectrum disorder; DEE = developmental and epileptic encephalopathy; ID = intellectual disability.

## Data Availability

Data are contained within the article.
